# Supplementary material for: The causal relationship between the human gut microbiota and pyogenic arthritis: a Mendelian randomization study
Source: Front Cell Infect Microbiol. 2024 Nov 26;14:1452480. doi: 10.3389/fcimb.2024.1452480 (PMC11629706; doi:10.3389/fcimb.2024.1452480)
Supplement: Supplementary file 1 [file DataSheet1.zip › mendelian test/ebi-a-GCST90027489.csv_forest.pdf]

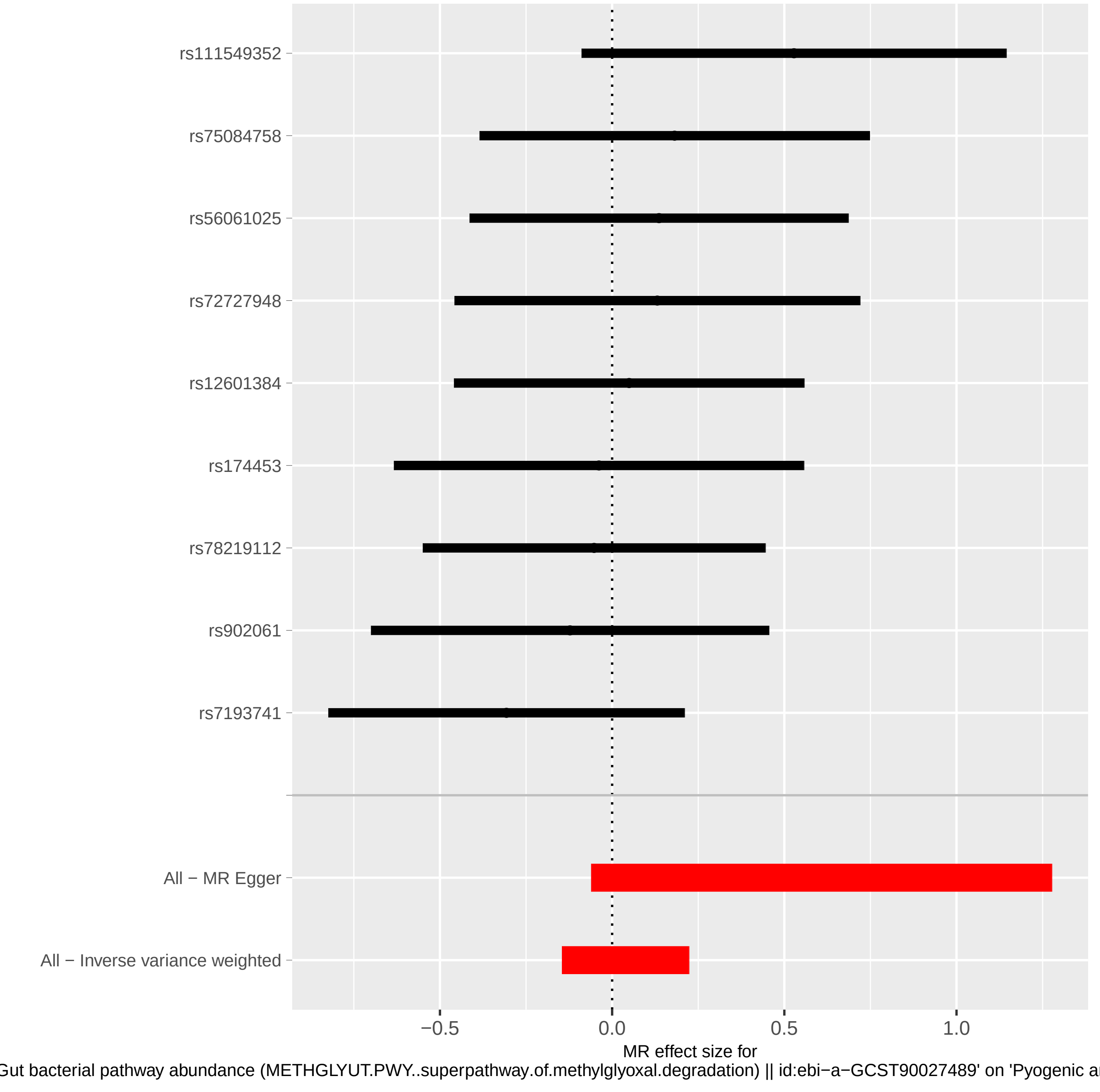

rs111549352
rs75084758
rs56061025
rs72727948
rs12601384
rs174453
rs78219112
rs902061
rs7193741
All − MR Egger
All − Inverse variance weighted
−0.5
0.0
0.5
1.0
MR effect size for
Gut bacterial pathway abundance (METHGLYUT.PWY..superpathway.of.methylglyoxal.degradation) || id:ebi−a−GCST90027489' on 'Pyogenic a
